# Gossypol acetate: A natural polyphenol derivative with antimicrobial activities against the essential cell division protein FtsZ

**DOI:** 10.3389/fmicb.2022.1080308

**Published:** 2023-01-12

**Authors:** Ruo-Lan Du, Ho-Yin Chow, Yu Wei Chen, Pak-Ho Chan, Richard A. Daniel, Kwok-Yin Wong

**Affiliations:** ^1^Department of Applied Biology and Chemical Technology and the State Key Laboratory of Chemical Biology and Drug Discovery, The Hong Kong Polytechnic University, Kowloon, Hong Kong SAR, China; ^2^Centre for Bacterial Cell Biology, Biosciences Institute, Faculty of Medical Sciences, Newcastle University, Newcastle upon Tyne, United Kingdom

**Keywords:** FtsZ inhibitor, antimicrobial activity, bacterial cell division, membrane permeability, synergistic effect

## Abstract

Antimicrobial resistance has attracted worldwide attention and remains an urgent issue to resolve. Discovery of novel compounds is regarded as one way to circumvent the development of resistance and increase the available treatment options. Gossypol is a natural polyphenolic aldehyde, and it has attracted increasing attention as a possible antibacterial drug. In this paper, we studied the antimicrobial properties (minimum inhibitory concentrations) of gossypol acetate against both Gram-positive and Gram-negative bacteria strains and dig up targets of gossypol acetate using *in vitro* assays, including studying its effects on functions (GTPase activity and polymerization) of Filamenting temperature sensitive mutant Z (FtsZ) and its interactions with FtsZ using isothermal titration calorimetry (ITC), and *in vivo* assays, including visualization of cell morphologies and proteins localizations using a microscope. Lastly, Bacterial membrane permeability changes were studied, and the cytotoxicity of gossypol acetate was determined. We also estimated the interactions of gossypol acetate with the promising target. We found that gossypol acetate can inhibit the growth of Gram-positive bacteria such as the model organism *Bacillus subtilis* and the pathogen *Staphylococcus aureus* [both methicillin-sensitive (MSSA) and methicillin-resistant (MRSA)]. In addition, gossypol acetate can also inhibit the growth of Gram-negative bacteria when the outer membrane is permeabilized by Polymyxin B nonapeptide (PMBN). Using a cell biological approach, we show that gossypol acetate affects cell division in bacteria by interfering with the assembly of the cell division FtsZ ring. Biochemical analysis shows that the GTPase activity of FtsZ was inhibited and polymerization of FtsZ was enhanced in vitro, consistent with the block to cell division in the bacteria tested. The binding mode of gossypol acetate in FtsZ was modeled using molecular docking and provides an understanding of the compound mode of action. The results point to gossypol (S2303) as a promising antimicrobial compound that inhibits cell division by affecting FtsZ polymerization and has potential to be developed into an effective antimicrobial drug by chemical modification to minimize its cytotoxic effects in eukaryotic cells that were identified in this work.

## 1. Introduction

Antimicrobial resistance (AMR) occurs when bacteria develop resistance or tolerance against antimicrobial medications. Infections by resistant strains are difficult to treat because with the efficiency of many existing antibiotics becomes compromised, the pool of effective drugs is diminishing. AMR has become a major global problem in recent years, with serious consequences for both public health and food safety. Hence, there is an urgent need for the discovery and development of new generations of antimicrobial drugs to tackle this serious public health problem. It is estimated that over half of the drugs used today are derived from or inspired by natural compounds, such as β-lactam antibiotics. Natural products made great contributions in the Golden Age of the discovery of antibiotics and they are still expected to contribute to solving the antibiotics crisis (Wright, 2017). Gossypol is a natural polyphenolic aldehyde from cotton plants and has many medical properties (Keshmiri-Neghab and Goliaei, 2014), such as anti-HIV/AIDS (Bourinbaiar and Lee-Huang, 1994), anticancer (Gilbert et al., 1995), antitumor (Xu et al., 2005), antivirus (Tai-Shun et al., 1993), antiparasitic (González-Garza et al., 1993), and antimicrobial properties (Margalith, 1967). Gossypol occurs as a mixture of two enantiomers, (+)- and (−)- gossypol, and the ratio between the two enantiomers alters its biological activities. Gossypol-acetic acid, a polyphenolic aldehyde, contains equal molar amounts of the (+)- and (−)-enantiomers (Gershenzon and Dudareva, 2007). Inhibitory effects of gossypol-acetic acid on *Edwardsiella ictalurid* have been reported before (Yildirim-Aksoy et al., 2004), but its inhibitory mechanism is not clear. Here, we studied the antimicrobial activity of gossypol acetate using model Gram-positive bacteria. The mode of action of gossypol acetate was studied *in vivo* and *in vitro* with the results showing that gossypol acetate very likely kills bacteria by directly targeting cell division.

Binary fission is the most common mode of bacterial reproduction (Angert, 2005), and it is carried out by a dynamic division machinery, the “divisome,” which consists of more than 10 essential division proteins (Kureisaite-Ciziene et al., 2018). Divisome is assembled on a scaffold (the *Z*-ring) (Du and Lutkenhaus, 2017) formed by the filament-forming homolog of tubulin, FtsZ, which is the first protein to be assembled at a division site and acts as the focal point for the recruitment of the downstream proteins (e.g., EzrA, PBP2B, and FtsA). FtsZ is essential for bacterial cell divisions and viability (Margolin, 2005; Lutkenhaus et al., 2012). Impairing FtsZ dynamic polymerizations can lead to the failure of divisome assembly, thus blocking cell division. Although cells can continue to grow in size initially, cell morphologies change to filamentous (from rods) or very large cocci (from cocci) and cells eventually lyse. FtsZ is a highly conserved protein among prokaryotes and is absent in higher eukaryote cells. Due to the essential and specific roles of FtsZ in bacterial cytokinesis and its conservation in prokaryotes, FtsZ represents an attractive target for developing new therapeutic antibiotics. A number of studies have been conducted to search for inhibitors of FtsZ and many compounds have been identified to have inhibitory effects on the functions of FtsZ (Tripathy and Sahu, 2019). One of the effective inhibitors is PC190723 (3-[(6-chloro[1,3]thiazolo[5,4-b]pyridin-2-yl)methoxy]-2,6-difluorobenzamide), and its binding site in FtsZ has been shown to be equivalent to the binding site of antitumor taxol in tubulin (Haydon et al., 2008). When seeking the target of gossypol acetate, we observed the elongation of *Bacillus subtilis* cells in the presence of gossypol acetate. Our data shows that gossypol acetate has inhibitory activity toward the functions of FtsZ, suggesting that FtsZ is very likely to be the main target of gossypol-acetic acid.

## 2. Materials and methods

### 2.1. Antimicrobial activities studies

Gossypol acetate (S2303) was purchased from Selleck (Huston, TX, USA) (99.78% purity). The minimum inhibitory concentrations (MIC) of S2303 against *B. subtilis* 168 (ATCC 23857), *Staphylococcus aureus* 259213, *Escherichia coli* 25922, and methicillin-resistant *S. aureus* strains (*S. aureus* BAA 41, *S. aureus* BAA 44, *S. aureus* ATCC 43300, *S. aureus* ATCC 33591, *S. aureus* ATCC 33592, and *S. aureus* BAA 1720) were determined according to the Clinical and Laboratory Standards Institute (CLSI) guidelines (Wikler, 2009). *S. aureus* strains were cultured in Cation adjusted Müller–Hinton Broth (CAMHB), whereas *B. subtilis* 168 and *E. coli* 25922 were cultured in Müller–Hinton Broth (MHB). The bacterial strains were inoculated into 5 mL of their corresponding media, and the cultures were grown overnight at 37°C with shaking at 250 rpm. The overnight cultures were diluted 10-fold into the fresh growth media and incubated in a rotary shaker for 2 h. After incubation, the absorbance of cell cultures at 600 nm (A_600_) was measured, and each culture was diluted to 5 × 10^6^ CFU/mL. Drug compounds were first dissolved in Dimethyl sulfoxide (DMSO) (12,800 μg/mL), followed by 2-fold serial dilutions. In a 96-well microplate, the concentration of DMSO in each well was maintained at 1% (v/v), and a 10-μL portion of the diluted cell culture was added into each well. After incubating the plate at 37°C for 16–18 h, the A_600_ of each well was measured using an iMark™ microplate reader (Bio-Rad, Hercules, CA, USA). This experiment was performed in duplicate.

### 2.2. Checkerboard assay

Synergistic effects of S2303 with Polymyxin B nonapeptide (PMBN) against *E. coli* 25922 were studied using a simplified checkerboard assay. S2303 (diluted by 2-fold) was added into a 96-well microplate with each well containing PMBN at the same concentration (20 μg/mL). Cell culture of *E. coli* 25922 with a cell density of 5 × 10^5^ CFU/mL was added into each well. After incubation at 37°C for 16–18 h with shaking, the A_600_ of each well was measured by an iMark™ microplate reader (Bio-Rad, Hercules, CA, USA). This assay was done in duplicate.

### 2.3. Time-kill assays

*Staphylococcus aureus* BAA 41 (5 × 10^5^ CFU/mL) was treated with S2303 at final concentrations of 1, 2, 4, and 8 times the MIC (8 μg/mL) in 3 mL of CAMHB. For the positive control, the cell culture was treated with 1% (v/v) DMSO. Aliquots of the treated cell culture were removed at the time points of 0, 1, 2, 4, 6, 8, and 24 h after the addition of S2303, and diluted to an appropriate concentration before spreading on Trypticase Soy Agar (TSA) in Petri dishes for CFU counts. After incubation for 24 h at 37°C, survived cells after treatment with S2303 or DMSO on Petri Dishes were counted, and time-kill curves of *S. aureus* BAA 41 of log CFU/mL were plotted against the time intervals. The minimum bactericidal concentration (MBC) of S2303 against *S. aureus* BAA 41 was defined as the lowest concentration required to reduce the initial bacterial counts by more than 99.9%. The experiment was repeated for three times.

### 2.4. Phase-contrast microscopic studies

Before treating with S2303 (2 μg/mL) or DMSO [1% (v/v)], the density of *B. subtilis* 168 was adjusted to A_600_ = 0.01. After treatment with S2303 or DMSO at 37°C for 4 h, cells were stained with 40 μM FM 4-64 dye [N-(3-Triethylammoniumpropyl)-4-[6-(4-(Diethylamino) Phenyl) Hexatrienyl] Pyridinium Dibromide] for 15 min at room temperature. A 1-μL portion of cells was then added onto a 1.2% agarose pad and covered with a cover glass. Morphologies of the cells were observed and imaged on a Ti2-E microscope (Nikon, New York, NY, USA) equipped with a CFI Plan Apo 60×/1.40 Oil objective by Nikon DS-Qi2 camera. For membrane imaging (stained by FM 4-64), FM 4-64 was excited at 550 ± 40 nm and the emission was collected at 630 ± 75 nm (49008, Chroma Technology, Olching, Germany). One hundred cells were randomly selected, and their cell lengths were measured with the ImageJ software (version 1.49). The experiment was repeated for three times.

### 2.5. Formation of *Z*-rings in *B. subtilis* cells

A derivative of the *B. subtilis* wild type strain 168 harboring a *ftsZ-gfp* fusion on a plasmid (Sun et al., 2017; Supplementary Table 2) was grown in Luria-Bertani (LB) broth at 37°C with 30 μg/mL chloramphenicol overnight. The overnight culture was diluted to A_600_ = 0.01 and diluted cell culture was treated with 2 μg/mL S2303 and 40 μM IPTG (Isopropyl β-D-1-thiogalactopyranoside) at 37°C with shaking for 4 h. The cell membranes were stained, and the live cells were examined and imaged as above (section “2.4. Phase-contrast microscopic studies”). For *Z*-ring imaging, FtsZ-GFP was excited at 470 ± 40 nm and the emission was collected at 525 ± 50 nm (49002, Chroma Technology, Olching, Germany).

### 2.6. GTPase activity assays

The *ftsZ* gene of *S. aureus* was cloned into the pRSET-A-S vector such that it was fused to a 6× His tag at the N-terminus. The overexpression plasmid was then introduced into *E. coli* BL21 (DE3) strain, 6× His-FtsZ protein was overexpressed and purified as described previously (Chan et al., 2013) and the procedures and the SDS-PAGE (Supplementary Figure 1) of purified *S. aureus* FtsZ can be found in Supplementary Data. The GTPase activity of SaFtsZ was measured on a Malachite Green Phosphate Assay Kit (Bio-Assay Systems, Hayward, CA, USA) (Amsbio, Online). SaFtsZ (3 μM) with S2303 at 2, 4, 8, and 16 μg/mL or 1% (v/v) DMSO, in 50 mM MOPS buffer (4-morpholinepropanesulfonic acid, pH 6.5) was incubated at room temperature for 30 min. MgCl_2_ (5 mM) and KCl (200 mM) were subsequently added into the reaction mixtures, and GTP (Guanosine-5′-triphosphate) hydrolysis was started by the addition of 250 μM GTP. A 80-μL portion of the reaction mixture was pipetted into each well of a clear bottom 96-wells microplate, followed by incubation at 37°C for 10 mins. A 20-μL portion of the working reagent was then added into each well. The plate was further incubated at 37°C for 30 min for color development. Released phosphates were quantified by measuring A_620_ using an iMark™ microplate reader (Bio-Rad, Hercules, CA, USA). The experiment was repeated for three times.’

### 2.7. Isothermal titration calorimetry studies

The thermodynamic properties of S2303 binding to SaFtsZ were analyzed by isothermal titration calorimetry (ITC) using a MicroCal PEAQ-ITC (Malvern Panalytical, Almelo, Netherlands) instrument. SaFtsZ was dialyzed against Tris–HCl buffer [20 mM Tris(hydroxymethyl)aminomethane hydrochloride, 150 mM NaCl, pH 7.5] with 20 times the volume of the protein solution for three cycles, and S2303 was dissolved in the same buffer. The concentrations of DMSO for both proteins in the cell and S2303 in the syringe were kept the same at 5% (v/v). A 0.4-μL portion of 200 μM S2303 was titrated with 20 μM SaFtsZ, and 300 s was given to maintain a zero-temperature difference between the reference and the sample cell for the first titration. Afterward, for every 4 s, a 2.0-μL portion of S2303 was titrated with SaFtsZ, and the spacing time was adjusted to 150 s. A total of 19 injections were done. Calculations of dissociation constant (*K*_*d*_), changes in Gibbs free energy (Δ*G*), enthalpies (Δ*H*), and entropies (Δ*S*) were carried out using the MircoCal PEAQ-ITC analysis software. The dissociation constant for the binding of S2303 to SaFtsZ was estimated by fitting the “One Set of Sites” mode into the MircoCal PEAQ-ITC analysis software.

### 2.8. 90° light scattering studies

A 90° light scattering assay was conducted to monitor the *in vitro* effect of S2303 on the assembly of *S. aureus* FtsZ filaments because the light scattering signal at 600 nm is proportional to the size of SaFtsZ polymer. Both excitation and emission wavelength were 600 nm, and the slit width was 2.5 nm. Before conducting a light scattering assay, the instrument (Cary Eclipse Fluorescence Spectrophotometer, Agilent, Santa Clara, CA, USA) was pre-warmed at 37°C. SaFtsZ [11 μM in 50 mM MOPS (3-(N-morpholino)propanesulfonic acid), pH 6.5)] was incubated at room temperature with S2303 for 10 min. KCl (50 mM) and MgCl_2_ (10 mM) were added to this mixture, and the whole mixture was then transferred to a quartz cuvette. Light scattering signals were recorded for 2,000 s after 1 mM GTP was added to the mixture.

### 2.9. Effects on the localization of the division proteins in *B. subtilis*

The strains used for studying the effects of S2303 on the assembly of the *B. subtilis* divisome expressed a WALP-mCherry fusion that was membrane-associated, therefore when these strains were used, membrane dye was not used. The division proteins examined in this experiment included EzrA (EzrA-GFP-WALP-mCh), FtsZ (FtsZ-GFP-WALP-mCh), and PBP2B (PBP2B-GFP-WALP-mCh). An FtsA-YFP-WALP-mCherry strain was constructed, but it was found to have a very weak red signal and so was not used in this analysis. The genotypes of these strains and the concentrations of the inducers used can be found in Supporting Information (Supplementary Tables 1, 2). The overnight culture of each strain was diluted 10 times in 1/5 LB medium which has been pre-warmed and supplemented with antibiotics and the corresponding inducer. After incubation for 2 h at 30°C, the cells were adjusted to OD_600_ = 0.05. A 1.0-mL portion of each prepared cell culture was treated with 1% (v/v) DMSO or 4 μg/mL S2303. After incubation for 10 mins at 37°C, cells were added to pads and examined as above. Images were taken using a Nikon Ti2-E microscope. For green fluorescence protein labeling proteins imaging, settings are same as in 2.5. For WALP-mCherry imaging, settings are same as FM 4-64 imaging. For FtsA-YFP imaging, FtsA-YFP was excited at 500 ± 20 nm and the emission was collected at 535 ± 30 nm (49003, Chroma Technology, Olching, Germany).

### 2.10. Measuring changes on the membrane turbidity

*Staphylococcus aureus* BAA 41 was inoculated into 5 mL of LB broth and cultured overnight with shaking at 37°C. Overnight cell culture was then washed by PBS for three times and the O.D. 600 was adjusted to 0.4. SYTOX green was added into the diluted cell suspension and the final concentration of SYTOX green is 5 μM. The SYTOX green/cell suspension was incubated at room temperature for 30 mins. Different concentrations of S2303 (2, 4, 8, 16, 32, 64, and 128 μg/mL) was added to 50 μL of PBS in a corning 96 flat black plate. 100 μg/mL Kanamycin and 0.5% DMSO was the negative control while 40 μg/mL Nisin (2.5%, Sigma, Marlborough, MA, USA) was regarded as the positive control. 50 μL cell suspension was added into each well. Fluorescence was measured at room temperature using a spectrophotometer with excavating and emission wavelengths of 485 and 525 nm, respectively. All experiments were conducted in triplicate.

### 2.11. *In vitro* hemolytic assays

Hemolytic effects of S2303 against erythrocytes were measured using a hemolysis test kit (HaemoScan, Groningen, Netherlands). All buffers used in this study were included in the kit. Frozen erythrocytes were initially recovered in an ice bath and then washed with 5 mL of the washing buffer and centrifuged at 4°C, 400× *g* (or 2,500 rpm) for 10 min. The supernatant was discarded after centrifugation, and the cells were washed repeatedly until the supernatant became clear pink. Afterward, erythrocytes were washed with 2 mL of the dilution buffer for 3 times. After washing, the dilution buffer was added to resuspend erythrocytes to obtain a final concentration of 5% erythrocytes. For each assay, a 100-μL portion of prepared erythrocytes was mixed with 100 μL of S2303 at the concentration of 0.5, 1, 2, 4, 8, 16, 24, 32, 48, 64, and 128 μg/mL [the final concentration of DMSO was 1% (v/v)]. Milli-Q water (100 μL) was added to 100 μL erythrocyte suspension as a negative control (0% hemolysis), while 100 μL 2% Triton X-100 was added to 100 μL erythrocyte suspension as a positive control (100% hemolysis). Milli-Q water with 2% DMSO (100 μL) was also mixed with same volume of RBCs suspensions. The mixtures were incubated at 37°C with shaking at 100 rpm for 1 h; the erythrocytes were centrifuged at 400× *g* for 10 mins before a 20-μL portion of the supernatant solution was mixed with 180 μL of the assay buffer and added to a 96-well microplate. The OD_415_ and OD_450_ values were measured using an iMark™ microplate reader (Bio-Rad, Hercules, CA, USA).

### 2.12. Cytotoxicity assays

The cytotoxicity of S2303 against both Detroit 551 (human normal skin cell) and Hep G2 (human liver cancer cell) were studied by MTT [3-(4,5-dimethylthiazol-2-yl)-2,5-diphenyl-2H-tetrazolium bromide] (Invitrogen) assays. The cells were counted and diluted to 5,000 cells in 100 μL of DMEM (Dulbecco’s Modified Eagle Medium) with 10% (v/v) FBS (Fetal bovine serum) and 100 U/ml penicillin and 100 U/ml streptomycin (All from Gibco except bovine calf serum which was from ATCC). Diluted cells were transferred to a 96-well tissue culture plate and grown overnight for cell adhesions onto the plate. Different concentrations (2.5, 5, 10, 20, and 40 μg/mL) of S2303 were added to the cells and the culture plate was incubated for up to 3 days. Then, the treated cells were mixed with 10 μL of 5 μg/mL water-soluble MTT regents, and the microplate was incubated at 37°C for a further 4 h. Only live cells could reduce MTT into purple formazan. After incubation, acidified SDS [0.01 N HCl in 10% (v/v) SDS (Sodium dodecyl sulfate)] was added to dissolve formazan, and absorbance at 570 nm was measured by a microplate reader. Cells treated with DMSO alone were used as the negative control. The cytotoxicity of S2303 against these two cell lines versus its concentrations was fitted into log (agonist) vs. response–variable slope in nonlinear regression (GraphPad Prism 8). The selective index was calculated as follows (Meletiadis et al., 2010):


$$\text{Selective}\,\text{index}\,\left(\text{SI}\right)=\frac{{\text{IC}}_{50}\,\text{μ}\,\text{g}/\text{mL}}{\text{MIC}\,\text{μ}\,\text{g}/\text{mL}}$$

### 2.13. Molecular docking studies

Molecular docking and analyses as well as observation of the possible binding mode of S2303 in *S. aureus* FtsZ (PDB code: 4DXD) were performed using CDOCKER with default parameter settings in BIOVIA Discovery Studio, 2016 (DS) (Studio, 2008). CDOCKER is an implementation of a CHARMm based docking tool using a rigid receptor. *S. aureus* FtsZ and S2303 were prepared using the “Prepared Protein” and “Prepared Ligands” modules in DS, respectively. A cleft located below the helix 7 and surrounded by the T7 loop and the four parallel β strands in *S. aureus* FtsZ was set as the binding site for docking, which is the same binding pocket of PC190723 in FtsZ (Haydon et al., 2008). Random conformations of S2303 were generated by high-temperature molecular dynamics with 1,000 dynamic steps at 1,000 K. Different conformations of S2303 were then docked into the binding site and the conformation orientations were subject to simulated annealing molecular dynamics with 2,000 steps heated to 700 K, and 5,000 steps cooled to 300 K. The CDOCKER energy or the CDOCKER interaction energy of final poses were calculated and ranked.

## 3. Results and discussion

### 3.1. S2303 has antibacterial activities

#### 3.1.1. S2303 (gossypol acetate) is able to exert antibacterial activity

Gossypol acetate shows inhibitory effects against *B. subtilis* 168 and *S. aureus* 29213 at 4 and 8 μg/mL, respectively; however, S2303 was unable to inhibit the growth of *E. coli* 25922 (Table 1). To test whether the inability of S2303 to kill *E. coli* 25922 was due to the outer membrane preventing S2303 from entering the cell, PMBN, an outer membrane permeabilizer (Dixon and Chopra, 1986), was used to increase the permeability of the outer membrane. As shown in Table 2, in the presence of 20 μg/mL PMBN, S2303 (64 μg/mL) was able to inhibit the growth of *E. coli*. The antimicrobial activities of S2303 against six methicillin-resistant *S. aureus* (MRSA) strains were also tested and are shown in Table 3; the MIC values of S2303 are 4–8 μg/mL, which are much lower than that of methicillin (256–1,024 μg/mL).

**TABLE 1:** Antimicrobial effects of S2303 against *Bacillus subtilis* 168, *Staphylococcus aureus* 29213, and *E. coli* 25922.

Compound	MIC (μg/mL)			Structure
	*B. subtilis* 168	*S. aureus* 29213	*E. coli* 25922	
S2303	4	8	>128	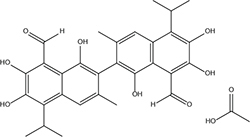

The chemical structure of S2303 is shown in this table. MIC, minimum inhibitory concentration.

**TABLE 2:** MIC of S2303 used in combination with Polymyxin B nonapeptide (PMBN) against *Escherichia coli* ATCC 25922.

Compounds	MIC (μg/mL)
	*E. coli* ATCC 25922
Polymyxin B nonapeptide (PMBN)	>256
S2303	>128
PMBN + S2303	20 + 64

**TABLE 3:** Activities of S2303 and methicillin against six MRSA strains.

MRSA Strains	MIC (μg/mL)	
	Methicillin	S2303
*S. aureus* BAA 41	1,024	8
*S. aureus* BAA 44	512	4
*S. aureus* ATCC 43300	256	8
*S. aureus* ATCC 33591	512	8
*S. aureus* ATCC 33592	256	8
*S. aureus* BAA 1720	256	8

MRSA, methicillin-resistant *Staphylococcus aureus* strains.

#### 3.1.2. Effect of S2303 on the growth of a typical MRSA strain over time

**FIGURE 1:**
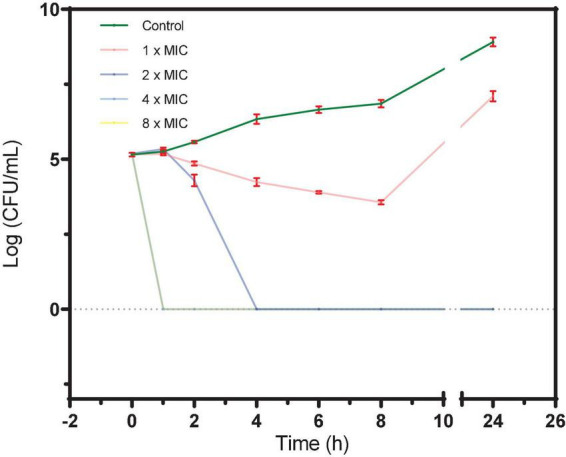
A time-kill curve of S2303 against *Staphylococcus aureus* ATCC BAA 41. *S. aureus* BAA 41 was grown in Cation adjusted Müller–Hinton Broth (CAMHB) and spread on Trypticase Soy Agar (TSA) plates for CFU counts. S2303 [1× MIC (pink), 2× MIC (dark blue), 4× MIC (light blue), and 8× MIC (yellow)] or Dimethyl sulfoxide (DMSO) [1% (v/v) (dark green)] was added to the cell cultures (5× 10^5 CFU/mL) at the start of the experiment (time zero), and the cultures were processed as described in the text. Time-kill curves of cells treated with 4× MIC (light blue) and 8× MIC (yellow) S2303 overlays and appear as a light green line in the plot. The MIC of S2303 against BAA 41 was 8 μg/mL. This result is representative of three independent experiments. Error bar is in color of red. When no cells grow, the number of cells is regarded as 1 for calculating log (CFU/mL) (=0).

As shown in Figure 1, after incubation of BAA 41 cells for 1 h with S2303 at 32 μg/mL (4× MIC) and 64 μg/mL (8× MIC), the optical densities of the cultures dropped to 0, indicating that most of the cells have been killed and lysed. At 16 μg/mL (2× MIC) S2303, all the bacterial cells were killed after incubation for 4 h. The MBC of S2303 against BAA 41 was estimated to be 16 μg/mL (2× MIC). Previous literature (Traczewski et al., 2009) has shown that the resistance of bacteria to an agent can be indicated by the ratio of MBC to MIC (resistance: ratio ≥ 32). In our case, the MBC-to-MIC ratio of S2303 against BAA 41 is 2, strongly suggesting that *S. aureus* BAA 41 is not resistant to S2303.

### 3.2. S2303 inhibits cell division by targeting FtsZ

#### 3.2.1. S2303 caused the elongation of *B. subtilis* 168 cells

**FIGURE 2:**
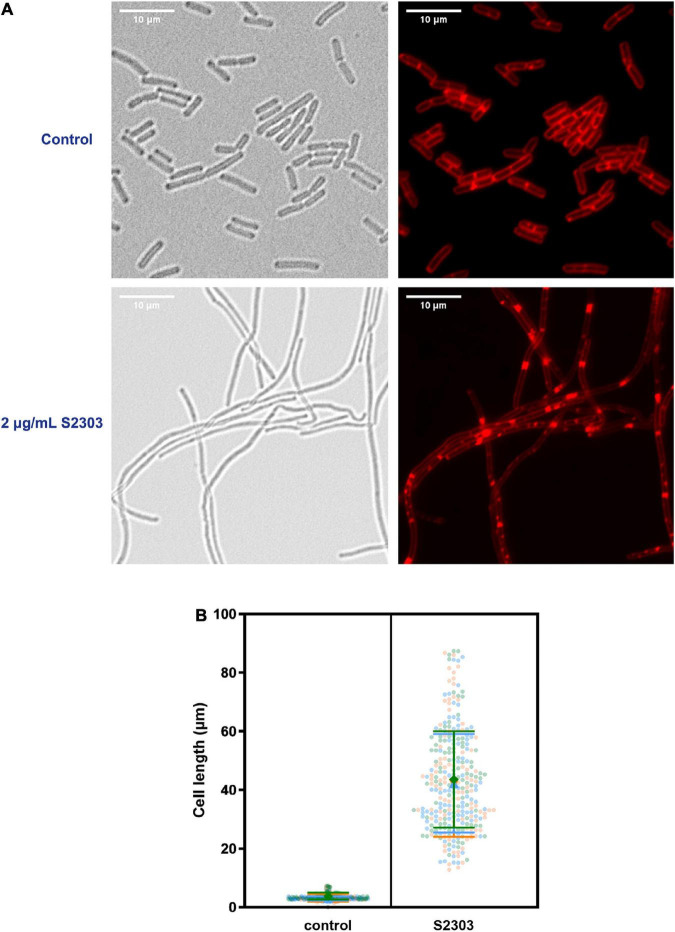
Elongation of *Bacillus subtilis* cell morphology was observed in cells treated by S2303. **(A)** Representative images of cells (OD_600_ = 0.01) treated with 1% (v/v) Dimethyl sulfoxide (DMSO) and 2 μg/mL S2303 were collected using a Nikon Ti2-E microscope. Cells were stained with FM 4-64 to mark the outline of the cells. There is a homogenous population of cells with short rod morphologies in cells treated with DMSO and a homogeneous population of cell filaments in cells treated with 2 μg/mL S2303. **(B)** The scale bar is 10 μm. In each of the three independent experiments, 100 cells of cells treated with 1% (v/v) DMSO and 2 μg/mL S2303 are randomly selected and measured using ImageJ and plotted using GraphPad. In each super plot, the middle large data points represent the median of the cell lengths of different replicates while two lines represent the corresponding standard deviation. The results of each experiment are plotted in a different color.

To investigate the mode of action of S2303, cells of *B. subtilis* 168 were treated with S2303 for 4 h and then stained with membrane dye before being examined by microscopy. Membrane staining allowed us to determine if the cells were filaments with no division or in chains, and also facilitated cell length measurement. While the control cells, treated with DMSO [1% (v/v)], appeared as normal short rods (Figure 2A), cells treated with 2 μg/mL S2303 became elongated and formed filaments and there was a homogeneous population of cell filaments in the group of cells treated with S2303 (Figure 2A). Cell lengths of 100 cells from both the control and the compound-treated cultures were measured, and violin plots of cell lengths were compared in Figure 2B. It is clear that the lengths of the treated cells were much longer when compared to the control, with an average cell length of 3.3 μm for the control cells and 42.6 μm for the treated cells (for 300 cells for both treated and untreated cells from 3 independent experiments). The results demonstrate that S2303 inhibits cell division, suggesting that it could be acting on one of the essential components of the cell division machinery. Considering FtsZ plays the fundamental role in divisome formation and cell division, FtsZ is the possible target and then the effects of S2303 on FtsZ were then studied. Besides, in those cells treated with S2303, septal membranes (stained by FM 4-64) between two daughter cells are thicker than that in control cells, indicating that cells encountered problems in separating cells. There also exist some spots in the cell membrane, suggesting that S2303 might also have some effects on bacterial membrane. Therefore, sides effects of S2303 were also studied.

#### 3.2.2. S2303 disturbed *Z*-ring formation

**FIGURE 3:**
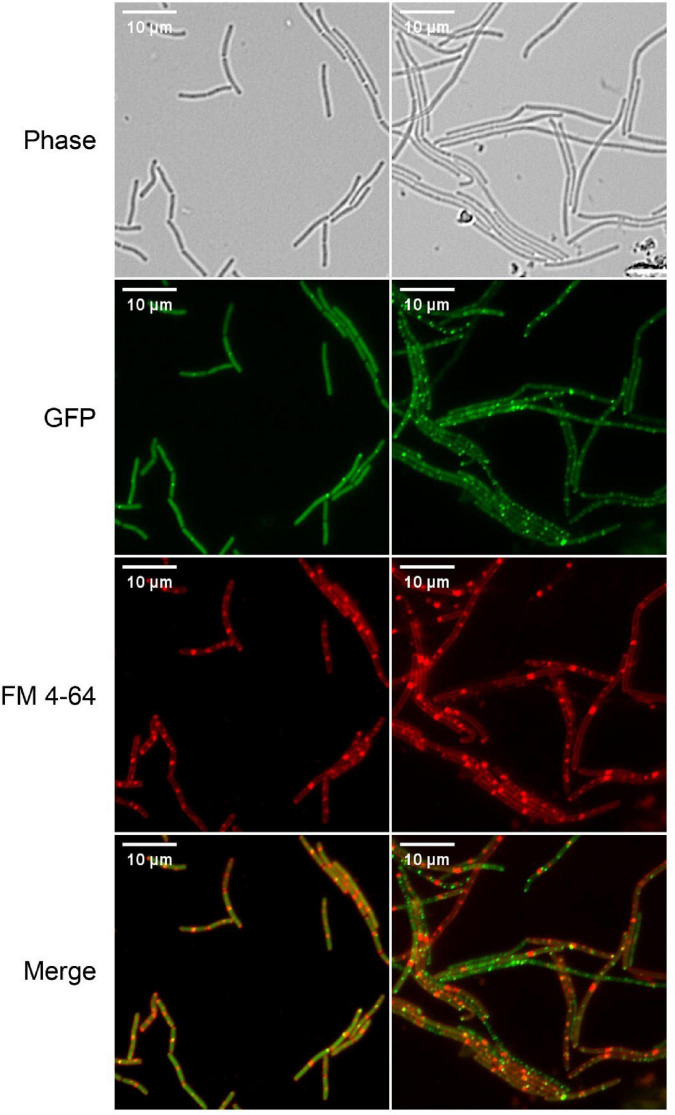
*Z*-ring is disturbed by S2303. Overnight culture of *Bacillus subtilis* 168 (harboring an inducible *ftsz-gfp* fusion on a plasmid) diluted in LB with inducer (40 μM IPTG) and were treated with 1% (v/v) Dimethyl sulfoxide (DMSO) or 2 μg/mL S2303 for 4 h. The expression of the ectopic copy of FtsZ-GFP was induced with 40 μM IPTG at 37°C. The cell membrane was stained by FM 4-64. The scale bar is 10 μm.

Correct localization of FtsZ and complete assembly of Z-ring and divisome are critical for cell division. So, the effect of S2303 on localizations of FtsZ was firstly examined using low level expression of an inducible FtsZ-GFP fusion, encoded on a plasmid, to allow detection FtsZ localization (Figure 3). In cells treated with 1% (v/v) DMSO for 4 h, as a control, FtsZ was seen as a general cytosolic signal in all cells and localization was predominantly seen at mid cell or polar positions in cell pairs. In contrast, when treated with S2303 FtsZ was seen to be more frequently in foci that did not correspond to potential sites of division. Comparing the merged images (merged red and green channel) of normal cells and cells treated with S2303, in control cells, most green spots overlayed with red spots indicating that FtsZ localized to the division site where septal membrane is forming or will form, while in cells treated with S2303 green spots appeared at many places where no red spots, indicating that FtsZ is delocalized and septal membrane formation is disturbed. The side effect of S2303 (4 h treatment), triggering red spots on cell membrane, were observed again. So, the cytotoxicity of the compound and its effects on membrane were investigated in section “3.5. Side effects and cytotoxicity of S2303.” And later microscopy experiments focused on short exposure to the compound to minimize the side effects of S2303. It was also evident that there was a significant level of cytosolic GFP signal in the cells analyzed in these experiments, potentially indicated that the FtsZ-GFP was overexpressed. To mitigate for this, further experiments used a chromosomally encoded inducible FtsZ-GFP (strain 2020) which permitted clearer localization of the FtsZ ring.

### 3.3. S2303 disturbs the assembly of divisome

#### 3.3.1. Delocalization of the other division proteins by S2303

The initial analysis showed that cell division was blocked in the strain treated with the compound, but since the strains were treated for a relatively long period, it was difficult to determine if the division defect was the primary mode of action for the compound, or if it was indirectly caused by the compound perturbing other aspects of cellular function. As the *Z*-ring structure plays a critical role in the formation of the divisome, serving as a scaffold for the divisome and recruiting the other cell division proteins. Disruption of *Z*-ring assembly by S2303 would be expected to result in the delocalization of the other division proteins after a relatively short period of treatment. Bacteria were then treated with S2303 for a shorter period (10 mins), and the localization of both FtsZ and proteins that were dependent on FtsZ, including the “early-localizing” EzrA and FtsA, and the “late localizing” PBP2B (Gamba et al., 2009) were examined in *B. subtilis* to find out which is the primary effects. Strains with chromosomally encoded EzrA, FtsA, FtsZ, and PBP2B labeled with fluorescent proteins (shown in Supplementary Table 2) were used for better visualization the effects of S2303. FtsA co-localizes with FtsZ and acts as the membrane anchor for FtsZ (Rico et al., 2004; Pichoff and Lutkenhaus, 2005; Krupka et al., 2017). EzrA is an accessory protein that modulates *Z*-ring formation (Levin et al., 1999; Haeusser et al., 2004; Haeusser Daniel et al., 2007), while PBP2B is a penicillin-binding protein that is essential for the synthesis of the septal wall (Errington et al., 2003).

**FIGURE 4:**
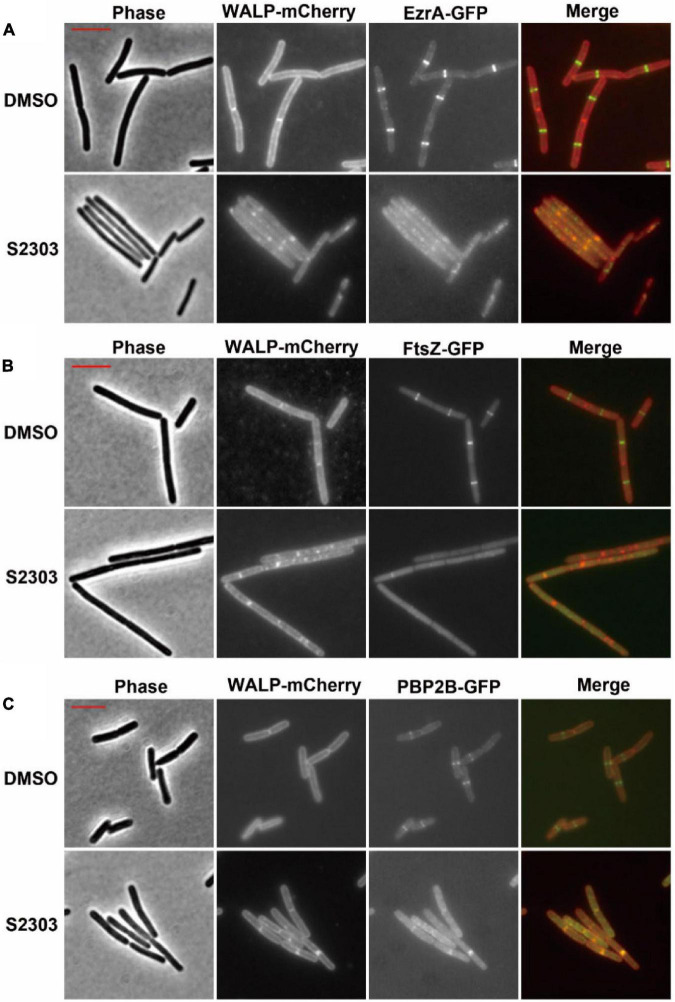
Effects of S2303 on the localizations of **(A)** EzrA, **(B)** FtsZ, and **(C)** PBP2B. The division proteins are fused to GFP (EzrA-GFP or PBP2B-GFP), while membranes are labeled by WALP-mCherry. In the merged images, membranes are shown in red and GFP in green. Cells (OD_600_ = 0.05) were incubated in 1/5 LB media with 4 μg/mL S2303 or Dimethyl sulfoxide (DMSO) [1% (v/v), “Untreated”] for 10 mins. The scale bar is 5 μm and in color of red.

In the control cultures treated with 1% (v/v) DMSO alone, clear localization of the proteins, appeared as bright fluorescent bands were seen at (potential) division sites in cells expressing EzrA-GFP, FtsZ-GFP, or PBP2B-GFP (Figures 4A–C, upper sets of images). Here the strains also expressed a membrane protein fused to the red fluorescent mCherry (WALP-mCherry) (Killian et al., 1996) that permitted simultaneous visualization of the cell membrane and so indicated the cell outline as well as the positions of the division septa. In contrast, when cells expressing EzrA-GFP and WALP-mCh were treated with 4 μg/mL S2303 for only 10 mins, EzrA-GFP was found to become dispersed, accompanied by a general increase in fluorescence in the whole cell (Figure 4A). In the red channel most of the cells did not have clear bands of membrane signal that corresponded to division septa. Instead, occasional patches of strong membrane signal could be seen inside the cell, at random positions. In *B. subtilis* FtsZ-GFP WALP-mCh cells, S2303 resulted in the green fluorescence signals of FtsZ to be reduced and dispersed over the cell, indicating that *Z*-rings were not formed (Figure 4B). Again, membrane signals corresponding to division septa were not present in these cells. Similarly, in most of the S2303 treated cells signals of PBP2B-GFP also became dispersed, but in some cells PBP2B remained localized (Figure 4C). However, these PBP bands always correspond to the bands of septal membrane, suggesting that at these sites the division septa were already well developed and probably had reached the stage of FtsZ ring disassembly, so are no longer sensitive to the compound. This would suggest that division had almost completed and it is probable that PBP2B localization is no longer dependent on FtsZ at this point in the process. However, it was clear that, in those affected daughter cells, PBP2B is delocalized compared to cells without treatment, suggesting that PBP2B localization will be affected when S2303 is added to treat those cells that have not finished the formation of divisome, since the platform is destroyed for PBP2B landing. These findings are consistent with the recently published journal (Whitley et al., 2021). Unfortunately, it was found that the combination of FtsA-YFP and WALP-mCherry did not permit useful co-localisation of the two fluorescent proteins. But examination of a strain expressing FtsA-YFP showed that S2303 also caused delocalization of FtsA-YFP (Supporting information, Supplementary Figure 2). Thus, the localization data is consistent with the rapid inhibition of the division process, by S2303, where FtsZ assembly would be expected to occur.

### 3.4. S2303 binds to FtsZ *in vitro* and inhibits the function of FtsZ

#### 3.4.1. S2303 inhibits the GTPase activity of FtsZ

**FIGURE 5:**
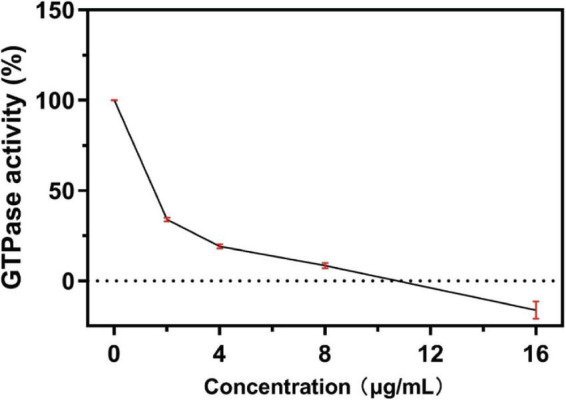
A concentration-response curve of S2303 against FtsZ GTPase activity. S2303 inhibits the *in vitro* GTPase activity of purified *S. aureus* FtsZ. Reactions contained 3 μM SaFtsZ, 50 mM MOPS pH 6.5, 5 mM MgCl_2_, 0.2 M KCl and 0.25 mM GTP and were incubated at 37°C for half an hour. Each point represents the average of three assays (*n* = 3) while red vertical bars show the standard error of the mean.

To understand the mechanism by which S2303 inhibited FtsZ ring assembly, we carried out *in vitro* analysis using purified FtsZ protein from *S. aureus* (SaFtsZ). SaFtsZ was successfully purified, and the purity was analyzed on SDS-PAGE (Supporting information, Supplementary Figure 1). As the GTPase activity is known to be important for the dynamic polymerizations of FtsZ (Mukherjee and Lutkenhaus, 1998), we first tested the effect of S2303 on the GTPase activity of FtsZ. Our results reveal that the GTPase activity of FtsZ was inhibited by S2303 in a dose-dependent manner (Figure 5); the GTPase activity of SaFtsZ was almost completely inhibited when FtsZ was pre-treated with 16 μg/mL S2303.

#### 3.4.2. S2303 stabilizes polymerizations of FtsZ

**FIGURE 6:**
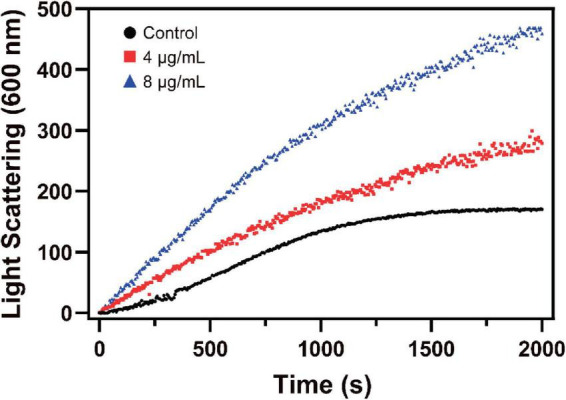
The effect of S2303 on *Staphylococcus aureus* FtsZ assembly *in vitro*. Polymerization of SaFtsZ (11 μM) was initiated by 1 mM GTP and the assembly of FtsZ in the absence [1% Dimethyl sulfoxide (DMSO), dark] or presence of 4 μg/mL (red) and 8 μg/mL (blue) S2303 was monitored by 90° angle light scattering. Appropriate blanks were subtracted from all the traces; the experiment was repeated in triplicate.

Changes in the assembly of FtsZ by S2303 were monitored by a 90° light scattering assay (Mukherjee and Lutkenhaus, 1998). As shown in Figure 6, the addition of S2303 into the FtsZ protein leads to an increase in the rate of light scattering signal in a dose-dependent manner, corresponding to a 1.75- and 2.80-fold increase of the light scattering signal in the presence of 4 and 8 μg/mL S2303, respectively, when compared with the control [1% (v/v) DMSO]. Our light scattering signal changes of FtsZ by PC190723, a known FtsZ inhibitor, can be found in Supplementary Figure 3. PC190723 stabilise protofilaments through its effect on GTPase activity in dose dependent manner. Thus, the effect of S2303 on the light scattering of FtsZ compared to PC190723 suggest that it may be acting in the same way. Literature also proved that PC190723 as a stabilizer of FtsZ (Andreu et al., 2010).

We speculated that S2303 binds to the active site of SaFtsZ and induces conformational changes of the protein such that the rate of SaFtsZ releasing GDP is reduced. It is possible that the GDP binding pocket of SaFtsZ becomes occupied as a result of the conformational change, which in turn reduces the overall rate of FtsZ hydrolysis and the release of SaFtsZ monomers from the bundles. This in some ways could be consistent with the microscopy results where S2303 initially causes FtsZ-GFP to be dispersed in the cells (Figure 3), but with time results in the formation of small aggregations of the FtsZ-GFP.

#### 3.4.3. Thermodynamic properties of the interaction

**FIGURE 7:**
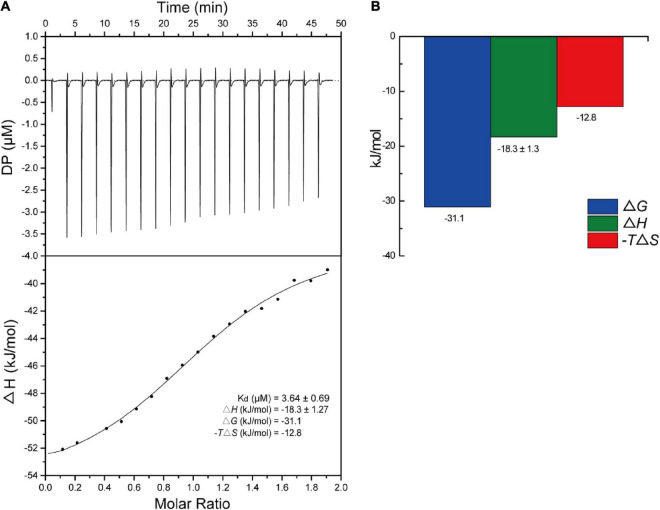
Titration of S2303 to SaFtsZ. **(A)** The raw isothermal titration calorimetry (ITC) data of S2303 binding to SaFtsZ (top) and the integrated heat peaks against the molar ratio of S2303 (bottom). **(B)** Thermodynamics properties of the S2303 and SaFtsZ interaction characterized by ITC. DP: a measured power differential between the reference and sample cells to maintain a zero temperature between them. The experiment was repeated in triplicate.

The binding interaction between S2303 and SaFtsZ was next studied using ITC (Figure 7). The equilibrium dissociation constant (*K*_*D*_) was found to be 3.6 ± 0.7 μM. Thermodynamic properties were calculated to understand the nature of the interactions between S2303 and SaFtsZ. Both the changes in enthalpies (Δ*H* = −18.3 ± 1.27 kcal/mol) and the contribution of changes in entropies (−TΔ*S* = −12.8) are favorable for binding, which reveals that the binding of S2303 to FtsZ is mainly contributed by hydrogen bonding and electronic interactions; the calculated difference in Gibbs free energy (Δ*G* = −31.1 kcal/mol) is negative, indicating that energies are released after their binding (Ross and Subramanian, 1981).

The binding of S2303 to FtsZ was proved by ITC and it was found to inhibit the GTPase activity of FtsZ and enhance and stabilize the polymerization of FtsZ. These results indicate that, in those cells treated with S2303, the *in vivo* functions of FtsZ, normal treadmilling and constriction, are disturbed by S2303. And FtsZ is the very potential target of S2303.

### 3.5. Side effects and cytotoxicity of S2303

#### 3.5.1. S2303 changes the membrane turbidity

**FIGURE 8:**
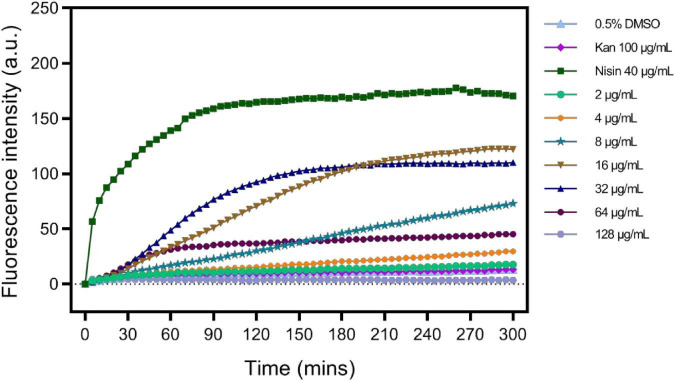
S2303 triggers the Membrane permeability changes of *Staphylococcus aureus* BAA 41. *S. aureus* BAA 41 was treated with S2303 at different concentrations. Dimethyl sulfoxide (DMSO) and kanamycin treatment are regarded as the negative control, while Nisin treatment is regarded as the positive control. Fluorescence intensity stands for the amount of SYTOX that has entered cells and binds to the nucleoid. Fluorescence intensity was measured by TECAN automated liquid handling system freedom EVO 100.

As in S2303 treated cells, fluorescence signals for membranes using both FM 4-64 and the WALP-mCherry appeared patchy (Figures 3, 5), suggesting that S2303 might affect the cell membrane and this might either be a secondary target of S2303 or an indirect effect of its inhibitory action on FtsZ. To test if S2303 was directly causing a change in membrane conformation, we analyzed membrane turbidity in the presence of different concentrations of S2303. A previous study has shown that SYTOX dye can act to discriminate between dead cells and living cells by flow cytometry (Yan et al., 2000). In our study, SYTOX dye was used to indicate a change in membrane permeability, as increased membrane permeability would allow the dye to enter the cells and thus stain the nucleoids brightly. Nisin was used as a positive control as it is a polycyclic antibacterial peptide that causes membrane permeability (Prince et al., 2016). Kanamycin, in contrast, was used as a negative control that inhibits cellular processes but does not directly affect the membrane. In the groups of cells treated with kanamycin and 0.5% DMSO, the fluorescence intensity changed very little, whereas for cells treated with Nisin the fluorescence intensity increased rapidly reaching a maximum after about 2 h (Figure 8). When cells were treated with different concentrations of S2303, the fluorescence intensity changes were detected, but were relatively small and slow to occur compared to that observed for nisin. It was also evident that at the concentrations used in the *in vivo* experiments there was some evidence of membrane damage, although at higher concentrations an intermediate effect was seen, with 32 μg/mL S2303 inducing the highest and most rapid increase in fluorescence intensity. Fluorescence intensity decreased at higher concentrations of S2303 (64 or 128 μg/mL). Surprisingly, higher concentrations triggered rapid cell death and potentially release and degradation of the DNA [as seen by Roth et al. (1997), Kim et al. (2019)] probably explaining the weaker fluorescence was observed at 64 and 128 μg/mL (Lebaron et al., 1998).

#### 3.5.2. S2303 induces little hemolysis of human blood cells

**FIGURE 9:**
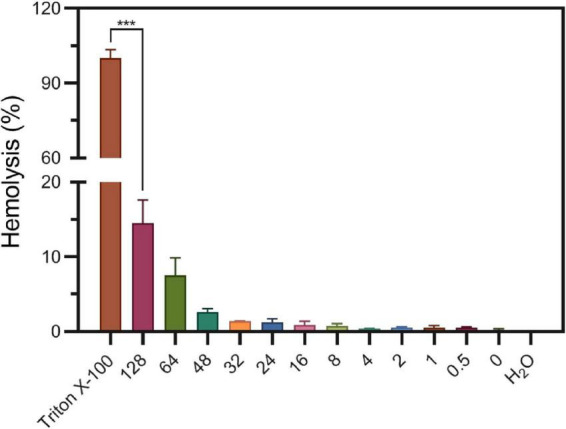
Hemolysis of S2303 on human blood cells. 1% Triton and H_2_O are regarded to trigger 100% hemolysis and 0% hemolysis, respectively. In other groups, RBCs were treated with different concentrations of S2303 (μg/mL). 0 μg/mL S2303 is equals to 1% Dimethyl sulfoxide (DMSO). *T*-test was checked between RBCs treated with 1% Triton X-100 and 128 μg/mL S2303 and *P*-value is less than 0.001 (***). Each point represents the average of data from three assays. The standard errors represent the error bars of the means.

Considering S2303 have side effects on bacterial membrane, triggering abnormal formation of cell membrane and membrane permeability changes, we worried S2303 will have toxicity on human cells. Thus, toxicity of S2303 on mammalian cells was studied. At first, the possibility of hemolytic effects of S2303 on human red blood cells (RBCs) was determined (Figure 9). Subjecting fresh erythrocytes to S2303 treatment at a range of concentrations showed minimal hemolysis, and only 14.5 ± 3.1% hemolysis occurred even at 128 μg/mL S2303. Importantly, at 4 μg/mL which is close to the minimum bactericidal concentrations of S2303 against methicillin-sensitive S. aureus (MSSA) and methicillin-resistant S. aureus (MRSA) strains (4–8 μg/mL), only 0.33 ± 0.09% RBCs lysed. Therefore, little hemolysis of RBCs was triggered by S2303.

#### 3.5.3. Cytotoxicity of S2303

**FIGURE 10:**
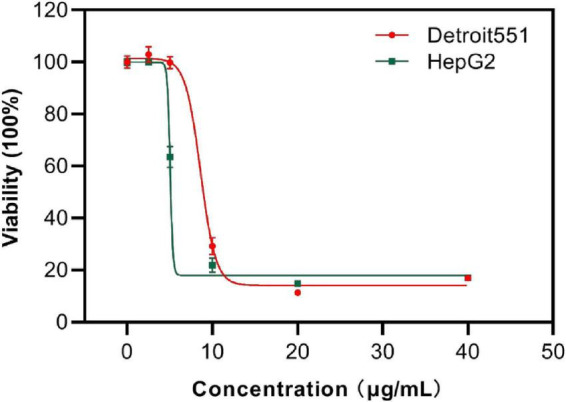
Cytotoxicity of S2303 against skin cells and liver cancer cells. Cytotoxicity of S2303 against skin cells and liver cancer cells. Detroit 551 is the human skin cell, and HepG 2 is the liver cancer cells, which are shown in red and green, respectively. Cells (5,000 per well) were seeded into 96-well plates and treated with S2303 of different concentrations for 24 h. Viability of cells was determined as a function of redox potential by MTT assay. Data were analyzed using GraphPad Prism 8.

Next, the viability of human skin cells (Detroit 551) and liver cancer cells (HepG 2) after exposure to S2303 were measured and evaluated as a dose-response curve. After a human skin cell line was treated with 8.80 μg/mL S2303, 50% of cells were killed; 90% of cells did not survive when the concentration of S2303 was increased to 12.2 μg/mL (Figure 10). Human liver cancer cells treated with S2303 exhibit IC_50_ = 6.6 μg/mL and IC_90_ = 16.3 μg/mL, respectively. As shown above, the minimum inhibitory concentrations of S2303 obtained from our antimicrobial activity studies were 4 μg/mL against *B. subtilis* 168 and 8 μg/mL against *S. aureus* strains.

The selective index (SI) (smaller SI, the more toxic the chemical is) of S2303 on the liver cancer cell line is 0.54, and the SI is 1.10–2.20 on resistant pathogens (MRSA), indicating that S2303 is very toxic. But the application of gossypol acetate as an antimicrobial agent might be better than its popular application in treating cancer because the SI as antimicrobial agent is larger.

Structural modifications on this compound to minimize its cytotoxicity is essential before further development as an antimicrobial agent. Chemical modifications on gossypol can be made on its hydroxyl groups, aldehyde groups and naphthalene rings (Lu et al., 2017; Liu et al., 2022). Some gossypol derivatives with high pharmacological activity and low toxicity have been synthesized by other research groups: gossypol Schiff bases with antifungal activity (Przybylski et al., 2009), antitumor agents (Dao et al., 2000, 2004), apogossypol against antiapoptotic Bcl-2 family proteins (Becattini et al., 2004), and so on. Gossypol-based antimicrobial agent discovery can lay a foundation for obtaining derivatives that might be suitable for medical and pharmaceutical applications as antimicrobial agents.

### 3.6. Molecular modeling studies

#### 3.6.1. Proposed binding mode

**FIGURE 11:**
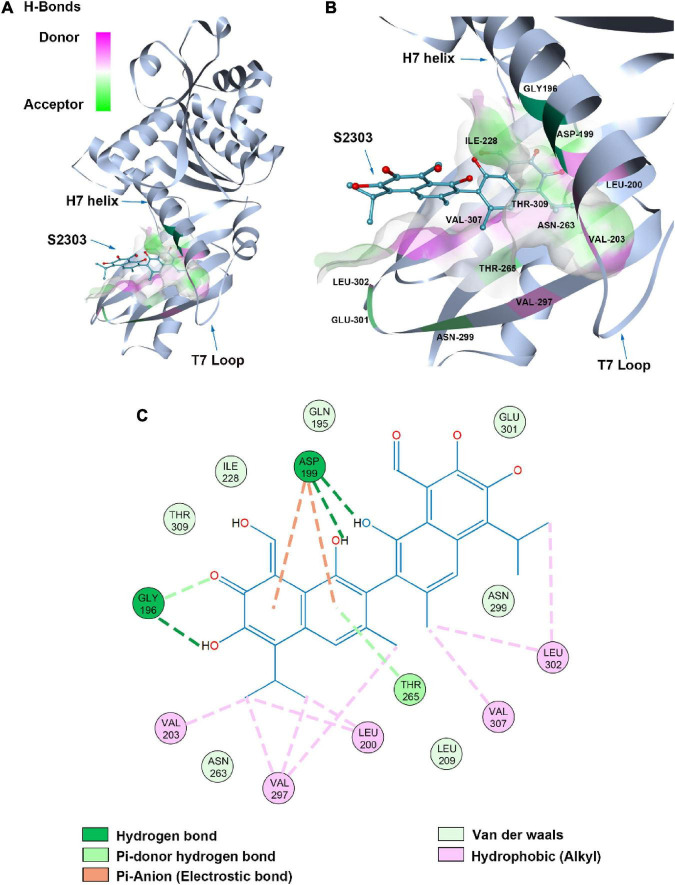
Proposed binding mode of S2303 in *Staphylococcus aureus* FtsZ (PDB code: 4DXD) analyzed by Discovery Studio (DS). The panel **(A)** shows the whole structure of FtsZ, and the binding pose of S2303 in the pocket while the panel **(B)** shows the enlarged hot spot, where we can see the amino acids which proposed have interactions with S2303. The 2D interaction diagram of S2303 with amino acids involved in the hot spot (PC190723 binding site in FtsZ) is shown in the panel **(C)**. The transparent interaction surface of FtsZ with S2303 is colored by hydrogen bond donor (red) or acceptor (green). Key amino acids estimated to participate in the interactions between S2303 and FtsZ on a 2D diagram are shown and labeled.

A proposed binding mode of S2303 in *S. aureus* FtsZ (PDB ID: 4DXD) was simulated and evaluated using the CDOCKER protocol in Discovery Studio (DS) (Figure 11), the CDOCKER energy and CDOCKER interactions energy are given by DS for ranking the calculated conformations of ligands in the crystal structure. Among the generated conformations of S2303, the lowest CDOCKER energies and CDOCKER interaction energies of this compound in FtsZ are 11.94 kcal/mol and −44.25 kcal/mol, respectively. The CDOCKER energies are the sum of interaction energies plus ligand energies (internal-ligand strain energy and receptor-ligand interaction energy), and the CDOCKER interaction energies are the interaction energies (receptor-ligand interaction energy) alone for each final binding mode (Rampogu and Rampogu Lemuel, 2016). In our proposed binding mode of S2303 in FtsZ, high CDOCKER energies (positive value) and low CDOCKER interaction energies of this binding mode were obtained (negative value)—this is because S2303 itself has higher energies and the proposed binding mode to the active site of FtsZ is energy favorable.

Gossypol acetate has a symmetrical structure containing two naphthalene groups. In our proposed binding mode of S2303 with FtsZ, one naphthalene inserts into the hydrophobic pocket formed by Val^203^, Val^297^, and Leu^200^. The side chain of Asp^199^ and the main chain near Gly^196^ are expected to have interactions with the hydroxyl groups on the naphthalene moiety through hydrogen bonding. Besides, Asp^199^ helps stabilize the inserted naphthalene group by π-anion interactions, which are relatively strong non-covalent interactions.

## 4. Conclusion

Based on our results, S2303 has an interesting potential as a starting point for the development of an antibacterial compound. This work indicates that gossypol acetate has the ability to interfere with the normal dynamic behavior of FtsZ *in vitro*, and this is supported by *in vivo* data. Gossypol acetate also increased membrane permeability over time, but the membrane disruption is not necessarily the cause of division inhibition. This is indicated by the fact that short incubation of cells with S2303 results in FtsZ delocalization, but has minimal effects on the membranes, something that is supported by other assays for membrane disruption (SYTOX staining and hemolysis of erythrocytes). So, this seems to be a long-term side effect of gossypol acetate. However, the membrane damaging activity is a problem for use of gossypol acetate as an antimicrobial agent and further structural modifications are needed to remove unwanted effects and enhance the specificity. This study highlights the major problems of drug development in that a compound that seems to have good specificity for the inhibition of an essential bacterial enzyme has a secondary activity that makes it unsuitable for systemic use, but might have application as a topical antibacterial agent. It is unclear at this point if modification of the compound to reduce its cytotoxic effects will reduce the antibacterial activity as it must have the ability to cross the membrane to interact with its target.

## Data availability statement

The original contributions presented in this study are included in this article/Supplementary material, further inquiries can be directed to the corresponding authors.

## Author contributions

R-LD: conceptualization, methodology, investigation, formal analysis, and writing—original draft. H-YC: performed the cytotoxicity experiment. YC: discussion and review and editing. P-HC: review and editing. RD: supervision and review and editing. K-YW: conceptualization, funding acquisition, resources, supervision, and writing—review and editing. All authors contributed to the article and approved the submitted version.
