# Supplementary material for: Gossypol acetate: A natural polyphenol derivative with antimicrobial activities against the essential cell division protein FtsZ
Source: Front Microbiol. 2023 Jan 12;13:1080308. doi: 10.3389/fmicb.2022.1080308 (PMC9878342; doi:10.3389/fmicb.2022.1080308)
Supplement: Supplementary file 1 [file Data_Sheet_1.docx]

Gossypol acetate: a natural derivative with antimicrobial activities against pathogenic bacteria by effective inhibition of FtsZ polymerizations and the subsequent assembly of divisomes for cell divisions

Ruo-Lan Du^1^, Ho-Yin Chow^1^, Yu-Wei Chen^1^, Pak-Ho Chan^1^, Richard Daniel^2^*, and Kwok-Yin Wong^1^*

^1^Department of Applied Biology and Chemical technology and the State Key Laboratory of Chemical Biology and Drug Discovery, The Hong Kong Polytechnic University, Kowloon, Hong Kong

^2^Centre for Bacterial Cell Biology, Biosciences Institute, Faculty of Medical Sciences, Newcastle University, Newcastle upon Tyne, United Kingdom

*** Correspondence:**Kwok-yin Wong
[kwok-yin.wong@polyu.edu.hk](mailto:kwok-yin.wong@polyu.edu.hk)

Richard Daniel
[richard.daniel@newcastle.ac.uk](mailto:richard.daniel@newcastle.ac.uk)

**Materials and Methods**

**Checkerboard assay**

The checkerboard assay was performed to study the synergistic effects between S2303 and methicillin (Odds, 2003). S2303 and methicillin were each diluted by 2-fold serial dilutions on a 96-well microplate. Each well contained a suspension of 5 x 10^5^ cells (MRSA strains) as well as S2303 and methicillin at different concentrations in 100 μL of CAMHB. After incubation for 16 - 18 h at 37°C, the A_600_ of each well was measured. This experiment was conducted in duplicate. The fractional concentration index (FICI) was calculated using the formula shown below (Hall et al., 1983):

$$\text{FICI=}\frac{\text{(MIC of agent A in a combination) }}{\text{MIC of agent A alone}}\text{+}\frac{\text{(MIC of agent B in a combination) }}{\text{MIC of agent B alone}}$$

FICI ≤ 0.5 indicates that there is a synergistic effect between the two agents; 0.5 < FICI ≤ 4 implies that the synergic effect is insignificant, whereas FICI > 4 shows “antagonism” (Meletiadis et al., 2010). Considering the error of the borderline FICI (0.5) caused by 2-fold serial dilutions, FICI around 0.5 is interpreted as a weak synergistic effect.

**Expression and purification of *S. aureus* FtsZ**

*E. coli* BL21 containing the pRSET-A-S vector with *S. aureus ftsz* was streaked out on a LB agar plate with 50 μg/mL ampicillin and incubated at 37°C overnight. Several colonies from the plate were inoculated into 5 mL of LB containing 50 μg/mL ampicillin and incubated with shaking at 250 rpm and 37°C overnight. 5 mL of the overnight culture was poured into 200 mL of 2 X TY supplement with 50 μg/mL ampicillin and incubated with shaking at 37°C until OD_600_ reached 0.8. Following that, IPTG was added to a final concentration of 0.4 mM to induce protein expression for another 4 hours at 37°C. After induction with IPTG, cells were centrifuged at 9000 rpm for 20 mins at 4°C, and the supernatant was discarded. The cell pellets can be stored at -80°C before purification steps.

The cells pellets were resuspended in 40 mL of the starting buffer (0.02M sodium phosphate, 0.5 M sodium chloride, pH 7.4) containing a half tablet of protease inhibitor. Then, cell disruption was carried out by a continuous high-pressure cell disruptor at 4°C to release the desired proteins inside of cells. Before use, the cell disruptor was washed by 250 mL deionized water twice, and then washed by 50 mL of the starting buffer at 10 psi and cooled to 4°C. Following that, the pressure was adjusted to 20 Kpsi, and the resuspended cell were placed in the cell disruptor, respectively. The soluble fractions and cell debris were separated by centrifugation at 10,000 rpm and 4°C for 2 hours. The supernatant was collected and filtered by sterile 0.22 μm syringe filters.

The soluble His_6_-SaFtsZ was purified by Fast Protein Liquid Chromatography (FPLC). A 5-mL HisTrap chelating column charged with nickel sulfate was used to capture His_6_-SaFtsZ. The column was equilibrated with the starting buffer (0.02 M sodium phosphate, 0.5 M sodium chloride, pH 7.4) before the supernatant was loaded on this column. After the proteins were loaded on the column, the unbound proteins were washed away. Following that, His_6_-FtsZ was eluted by increasing the percentage of elution buffer (0.02 M sodium phosphate, 0.5 M sodium chloride, 500 mM imidazole, pH 7.4).

Eluted samples were collected and loaded onto a 5 mL HiTrap Q HP column (GE Healthcare). FtsZ was eluded with a 750 mM gradient (50 mM MOPS, pH 6.5), and purity of FtsZ was then analyzed by 12% SDS-PAGE. The buffer of FtsZ was exchanged to 50 mM MOPS (pH 7.5).

**Results**

**No significant synergistic effects between S2303 and methicillin**

Fractional inhibitory concentration indexes (FICIs) of S2303 with methicillin (Meth) were measured to study their synergistic effects (**Table S1**). The FICIs of S2303 with methicillin against the three MRSA strains were around 0.5, indicating that S2303 has no synergistic effect with methicillin.

**Table S1 Synergistic effects of S2303 with methicillin (meth) against MRSA strains.** FICI: Fractional inhibition concentration index. The formula for calculating FICI is shown in the text.

|  | **MRSA Strains** | | |
| --- | --- | --- | --- |
|  | BAA 41 | BAA 1720 | ATCC 33591 |
| MIC of Meth (μg/mL) | 1024 | 256 | 512 |
| MIC of S2303 (μg/mL) | 8 | 8 | 8 |
| MIC of (Meth + S2303) (μg/mL) | 16 + 4 | 4 + 4 | 4 + 4 |
| FICI | 0.516 | 0.516 | 0.508 |

**Table S2** Genotypes of the constructed *B. subtilis* strains. Spectinomycin and chloramphenicol were used as selective antibiotics for the four strains. FtsA-YFP-WALP-mCh was not successfully constructed as the resulting strain showed very weak red signals in *B. subtilis.* * for strain constructions donor genomic DNA was transformed (→) into a specific recipient selecting for the appropriate introduced antibiotic resistance marker.

| ***B. subtilis* Strains** | **Genotypes** | **Reference** |
| --- | --- | --- |
| BS-FtsZ-gfp | *B. subtilis* 168 with a plasmid: pFZ-FtsZ-eGFP | (Patent No. CN105461585A, 2016) |
| 3312 | *trpC2 ezrA-gfp-cat* | (Gamba et al., 2009) |
| 2020 | *trpC2 amyE::Pxyl- gfp-ftsZ cat* | (J Sievers, unpublished) |
| 3122 | *trpC2 pbpB::pSG5061 (cat Pxyl-gfp-pbpB)* | (Scheffers et al., 2004) |
| PG62 | *trpC2 aprE*::P*_spac_-yfp-ftsA spec* | (Scheffers et al., 2004) |
| WALP-mCh | *trpC2 aprE::WALP-mCh spec* | (S, Lee, Unpublished) |
| EzrA-GFP-WALP-mCh | *trpC2 ezrA-gfp-cat aprE::WALP-mCh spec* | 3312 gDNA → WALP-mCh * |
| FtsZ-GFP-WALP-mCh | *trpC2 amyE::Pxyl-ftsZ-gfp cat aprE::WALP-mCh spec* | 2020 gDNA → WALP-mCh |
| PBP2B-GFP-WALP-mCh | *trpC2 amyE::Pxyl-pbpB-gfp cat aprE::WALP-mCh spec* | 3122 gDNA → WALP-mCh |

**Table S3 Concentrations of the inducers and antibiotics for the listed constructed *B*. *subtilis* strains.** Cm and Spec are abbreviation of chloramphenicol and spectinomycin, respectively.

| ***B. subtilis* Strains** | **Inducer** | **Antibiotics** |
| --- | --- | --- |
| EzrA-GFP-WALP-mCh | / | 60 μg/mL Spec and 5 μg/mL Cm |
| FtsZ-GFP-WALP-mCh | 0.1% xylose |  |
| PBP2B-GFP-WALP-mCh | 1% xylose |  |
| FtsA-YFP | 0.5 mM IPTG | 60 μg/mL Spec |


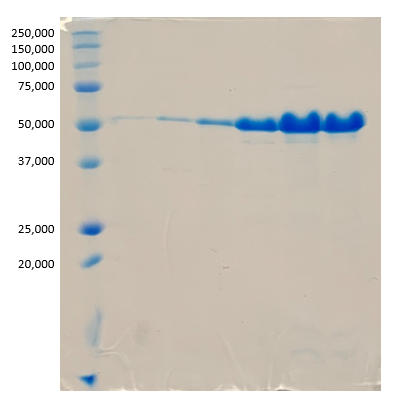


**Figure S1 SDS-PAGE of SaFtsZ.** From left to right, the first lane is the marker and the molecular weight of each band was labeled on the image. Other lanes are the fractions of elutions of SaFtsZ.


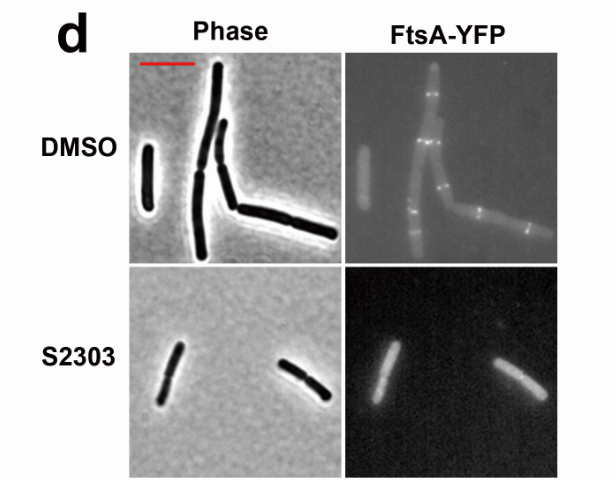


**Figure S2 Effect of S2303 on the localization of FtsA.** The green fluorescence arises from the polymer of FtsA-YFP. Cells were incubated in 1/5 LB media with 4 μg/mL S2303 or DMSO (1% (v/v), for 10 mins. The scale bar is 5 μm.

**
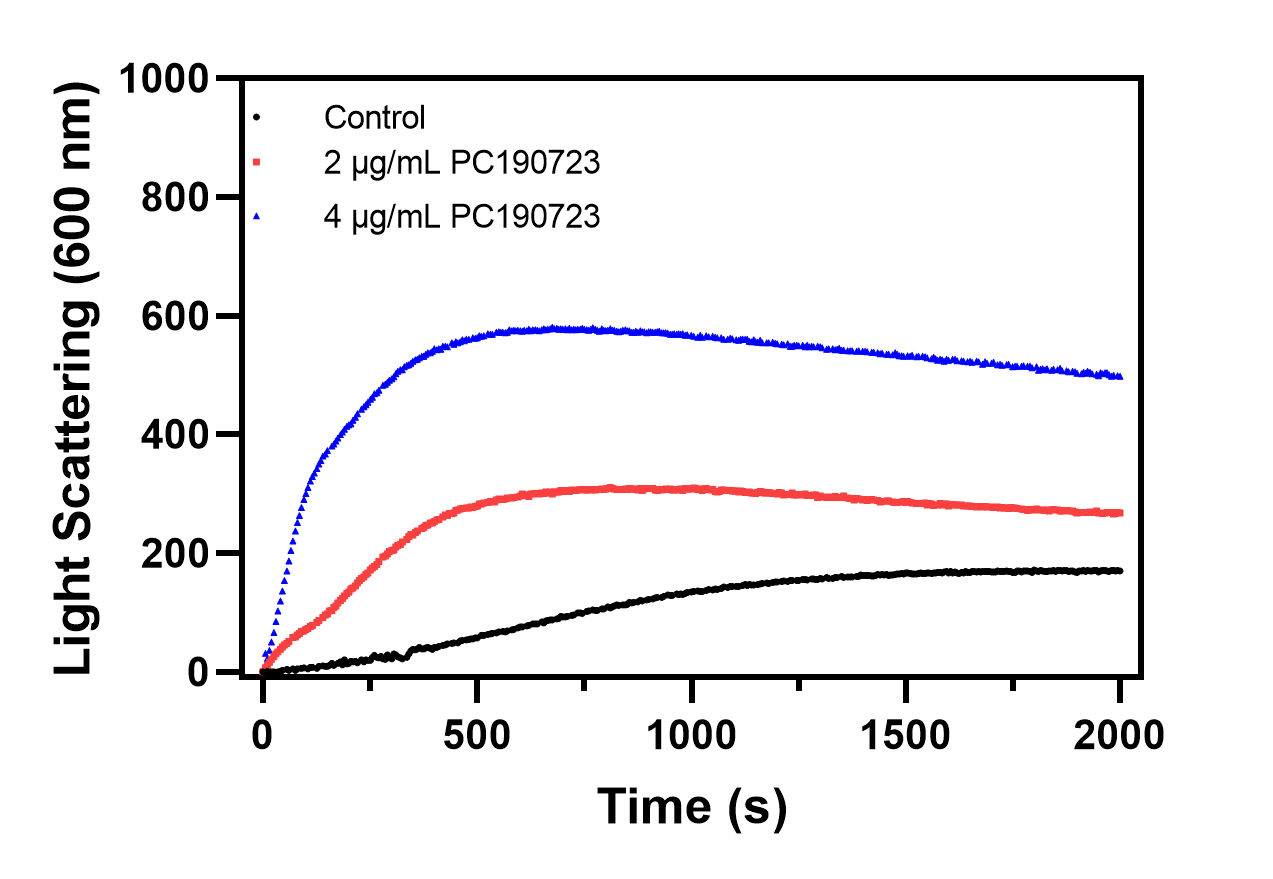
**

**Figure S3 The effect of PC190723 on *S. aureus* FtsZ assembly *in vitro*.** Polymerization of SaFtsZ (11 μM) was initiated by 1 mM GTP and the assembly of FtsZ in the absence (1% DMSO, dark) or presence of 2 μg/mL (red) and 4 μg/mL (blue) PC190723 was monitored by 90° angle light scattering. Appropriate blanks were subtracted from all the traces; the experiment was repeated in triplicate.

**References**

Gamba, P., Veening, J.-W., Saunders Nigel, J., Hamoen Leendert, W., and Daniel Richard, A. (2009). Two-Step Assembly Dynamics of the Bacillussubtilis Divisome. *J. Bacteriol.* 191(13)**,** 4186-4194. doi: 10.1128/JB.01758-08.

Hall, M.J., Middleton, R.F., and Westmacott, D. (1983). The fractional inhibitory concentration (FIC) index as a measure of synergy. *J. Antimicrob. Chemother.* 11(5)**,** 427-433. doi: 10.1093/jac/11.5.427.

Meletiadis, J., Pournaras, S., Roilides, E., and Walsh, T.J. (2010). Defining fractional inhibitory concentration index cutoffs for additive interactions based on self-drug additive combinations, monte carlo simulation analysis, and *in vitro*-*in vivo* correlation data for antifungal drug combinations against aspergillus fumigatus. *Antimicrob. Agents Chemother.* 54(2)**,** 602-609. doi: 10.1128/AAC.00999-09.

Odds, F.C. (2003). Synergy, antagonism, and what the chequerboard puts between them. *J. Antimicrob. Chemother.* 52(1)**,** 1-1. doi: 10.1093/jac/dkg301.

Scheffers, D.-J., Jones, L.J.F., and Errington, J. (2004). Several distinct localization patterns for penicillin-binding proteins in Bacillus subtilis. *Mol. Microbiol.* 51(3)**,** 749-764. doi: 10.1046/j.1365-2958.2003.03854.x.
